# Think Hard or Think Smart: Network Reconfigurations After Divergent Thinking Associate With Creativity Performance

**DOI:** 10.3389/fnhum.2020.571118

**Published:** 2020-11-20

**Authors:** Hong-Yi Wu, Bo-Cheng Kuo, Chih-Mao Huang, Pei-Jung Tsai, Ai-Ling Hsu, Li-Ming Hsu, Chi-Yun Liu, Jyh-Horng Chen, Changwei W. Wu

**Affiliations:** ^1^Graduate Institute of Biomedical Electronics and Bioinformatics, National Taiwan University, Taipei, Taiwan; ^2^Department of Psychology, National Taiwan University, Taipei, Taiwan; ^3^Department of Biological Science and Technology, National Chiao Tung University, Hsinchu, Taiwan; ^4^Intramural Research Program, Neuroimaging Research Branch, National Institute on Drug Abuse, National Institutes of Health, Baltimore, MLD, United States; ^5^Department of Radiology, Wan Fang Hospital, Taipei Medical University, Taipei, Taiwan; ^6^Department of Radiology, School of Medicine, College of Medicine, Taipei Medical University, Taipei, Taiwan; ^7^Department of Radiology and Brain Research Imaging Center, University of North Carolina at Chapel Hill, Chapel Hill, NC, United States; ^8^Graduate Institute of Mind, Brain, and Consciousness, Taipei Medical University, Taipei, Taiwan; ^9^Brain and Consciousness Research Center, Taipei Medical University-Shuang-Ho Hospital, New Taipei, Taiwan

**Keywords:** creativity, divergent thinking, functional connectivity, resting-state fMRI, alternative usage task (AUT), creative achievement questionnaire (CAQ)

## Abstract

Evidence suggests divergent thinking is the cognitive basis of creative thoughts. Neuroimaging literature using resting-state functional connectivity (RSFC) has revealed network reorganizations during divergent thinking. Recent studies have revealed the changes of network organizations when performing creativity tasks, but such brain reconfigurations may be prolonged after task and be modulated by the trait of creativity. To investigate the dynamic reconfiguration, 40 young participants were recruited to perform consecutive Alternative Uses Tasks (AUTs) for divergent thinking and two resting-state scans (before and after AUT) were used for mapping the brain reorganizations after AUT. We split participants into high- and low-creative groups based on creative achievement questionnaire (CAQ) and targeted on reconfigurations of the two brain networks: (1) default-mode network (DMN) and (2) the network seeded at the left inferior frontal gyrus (IFG) because the between-group difference of AUT-induced brain activation located at the left IFG. The changes of post-AUT RSFCs (DMN and IFGN) indicated the prolonged effect of divergent thinking. More specifically, the alterations of RSFC_IFG−AG_ and RSFC_IFG−IPL_ (AG: angular gyrus, IPG: inferior parietal lobule) in the high-creative group had positive relationship with their AUT performances (originality and fluency), but not found in the low-creative group. Furthermore, the RSFC changes of DMN did not present significant relationships with AUT performances. The findings not only confirmed the possibility of brain dynamic reconfiguration following divergent thinking, but also suggested the distinct IFGN reconfiguration between individuals with different creativity levels.

## Introduction

Creativity is the foundation of originality, the generation of novel ideas when facing a specific problem (Sternberg, 1999; Runco and Jaeger, 2012), and the cornerstone of productivity in human civilization and modern society. However, despite the current surging importance of creativity, its manifestations in the brain involves a complex architecture, the underlying mechanisms of which require an extensive investigation to disentangle (Sternberg and Lubart, 1996). The current psychometric creativity measures are largely based on Guilford's theory, according to which creative people have high ideational fluency and high degrees of novelty (Guilford, 1967). To date, the understanding of the internal process of creativity tended to alternate between the generation of novel ideas (i.e., divergent thinking) and the evaluation of generated ideas (i.e., convergent thinking). In the dual-process conception of creativity (Abraham, 2013; Sowden et al., 2015), divergent thinking represents the acquisition of a certain task and diverts attention away from the task itself, in a highly spontaneous manner to generate ideas, whereas the convergent thinking is associated with deliberate constraints and the verification of illuminated ideas (Christoff et al., 2016). Therefore, the divergent thinking can be regarded as an imperative incubation step before the “eureka!” moment reaches the mind. More importantly, the divergent thinking ability has moderate potential to predict creative achievements in the real world (Plucker, 1999). Based on the operational definitions of creativity, scientific disciplines have endeavored to unveil the sophisticated and fascinating mental processes of divergent thinking in the human brain.

In the neuroscience of creativity, questionnaires and cognitive tasks, such as creative achievement questionnaire (CAQ) for individual creativity achievements and alternative uses task (AUT) or the Torrance Tests of Creative Thinking (TTCT) for divergent thinking, were developed to probe the underlying mental processes of creativity (Plucker, 1999; Sternberg, 1999; Carson et al., 2005). With the support of neuroimaging technologies, the neural substrates of divergent thinking have been progressively disclosed in the literature, although with great diversity across brain regions. For example, Jung et al. discovered that the CAQ was positively correlated with the left lateral orbitofrontal volume and the cortical thickness of the right angular gyrus (Jung et al., 2010). Ellamil et al. demonstrated the involvement of the medial temporal lobe in a creative drawing generation task (Ellamil et al., 2012). Using the AUT, Fink et al. demonstrated that originality was positively associated with the activation of the temporal-parietal junction, medial prefrontal cortex (mPFC) and posterior cingulate cortex (PCC) (Fink et al., 2010). Benedek et al. concluded that the left inferior parietal cortex and left prefrontal regions subserved the flexible integration of previous knowledge for constructing novel and creative ideas (Benedek et al., 2014). Abraham et al. demonstrated that the left inferior frontal gyrus (IFG) and temporal poles played major roles in AUT engagements (Abraham et al., 2012). Moreover, a meta-analysis study revealed that the left IFG was among the most predominantly activated regions associated with idea generation across task-induced brain regions (Gonen-Yaacovi et al., 2013), suggesting its functional role in the semantic processing and conceptual expansion to expand the acquired concept into novel elements (Ward, 1994; Abraham, 2014; Boccia et al., 2015). In sum, emerging evidence indicates that brain regions in the left IFG, mPFC, parietal lobe, and medial temporal lobe are potentially involved in the mental process of divergent thinking.

Beyond the perspective of creative-task induction, divergent thinking can be taken as a spontaneous-thought process for idea incubation because of its involvement in the dynamic shifts between memory, emotion and attention (Sowden et al., 2015; Christoff et al., 2016). Therefore, a growing body of creativity-related neuroimaging studies, targeting the mental process of divergent thinking, have moved toward uncovering the long-distance brain connections using resting-state functional magnetic resonance imaging (rs-fMRI) (Takeuchi et al., 2012; Beaty et al., 2014; Wei et al., 2014). The default-mode network (DMN) has been determined to play an active role in idea generation, and the executive network appears to support idea evaluation instead (Jung et al., 2013; Beaty et al., 2016; Shi et al., 2018). Interestingly, the involvements of DMN connectivity in divergent thinking accords with the findings concerning task engagements, except for the left IFG. By separating participants into high- and low-creative groups based on their creativity score, Beaty et al. revealed greater connectivity strengths between the left IFG and posterior DMN in the high-creative group in contrast to the low-creative group (Beaty et al., 2014), which implies that the creative abilities lead to the variability of brain functionality. Recently, the DMN was further identified as a member of high-creativity neural circuits using connectome-based predictive modeling and AUT-based fMRI datasets (Beaty et al., 2018). The same study also inferred the plausible dynamicity of the network organization after divergent thinking by revealing the higher predictive power of a high-creativity network on the creativity score during AUT, compared with that under normal resting conditions. Wei et al. further suggested the possibility of dynamic resting-state functional connectivity (RSFC) changes by demonstrating that the low-creative group exhibited stronger RSFC changes between the mPFC and the middle temporal gyrus (MTG) after a creativity-related training task (Wei et al., 2014). Given the dynamic nature of creative thinking, it is speculated that the integrity of creativity-associated brain networks changes dynamically following the divergent thinking. However, are the dynamic network reconfiguration associated with the creativity performance? Do different creativity levels lead to distinct RSFC alterations? These unanswered questions are further addressed to better understand the brain functionality underlying creativity.

Herein, we establish the following hypotheses: (1) AUT-induced RSFC changes are associated with the creativity performances, and (2) individuals with different levels of creativity use distinct strategy of brain reconfigurations to comply with the challenges of AUT. To test these hypotheses, we recruited forty young participants to perform AUTs and compared their RSFC maps between two rs-fMRI sessions (Pre- and Post-AUT). The levels of creativity were assessed through splitting the participants into two groups (high- and low-creative) based on the CAQ scores and we tested the influence of AUT on RSFC changes for each creativity group.

## Materials and Methods

### Participants

A total of 42 right-handed healthy young adults were recruited in this study. The inclusion criteria were as follows: (a) non-smoking and without drug addiction; (b) not pregnant; (c) no history of neurological and psychiatric disorder history, and (d) no metal body implants. The entire protocol was approved by the Research Ethics Committee of National Taiwan University (NTU-REC No. 201407EM028). Because two participants could not complete the entire experimental procedure, their datasets were excluded in the analysis. Accordingly, the following results are reported for the remaining 40 participants (19 males, mean age = 24.4 ± 2.8 years, range: 21–33 years).

### Stimuli of AUT

The stimuli consisted of 56 grayscale images of objects from daily life with background removed. Prior to the actual fMRI experiment, we rated the number of alternative usages for each object stimulus from a separate group of participants (*N* = 15, 12 males, mean age = 23.4 ± 3 years, range: 20–30 years). During the rating, the 56 objects were presented consecutively to the participants with inter-trial intervals (ITIs) of 26 s. The participants were instructed to think and express alternative usages for each object within 20 s. The mean number of answer for each object was 2.4. We categorized the difficulty of AUT into three levels as follows: normal, 1.99–2.99 answers per picture; difficult, <1.99 answers per picture; and easy, >2.99 answers per picture. Subsequently, we used the task materials to design the AUT in MRI environments. All of the objects were presented against a white background for both rating and fMRI experiment.

### Experimental Procedure

After consenting, the participants were instructed to complete a CAQ, containing eight aspects of creativity (Carson et al., 2005), and to undergo the fMRI experiments. The fMRI experiment included seven sessions in total: two resting-state, two 0-back and three AUT sessions. The participants underwent the first rs-fMRI scan (Pre-AUT), performed three consecutive AUTs with two 0-back tasks in between, and went through the second rs-fMRI scan (Post-AUT) at the end. The participants only received the instruction to generate novel ideas after viewing pictures in the AUT sessions without practices to ensure the Pre-AUT resting state was irrelevant to divergent thinking. The multiple AUTs were designed to enhance the divergent thinking process with undemanding tasks (0-back) in between (Baird et al., 2012). Herein, we considered all AUTs and 0-backs as instructed divergent-thinking engagements, and adopted the two sessions of resting-state scans (Pre-AUT and Post-AUT) to discern the alterations of RSFC through the divergent thinking process.

### fMRI Acquisition and Scanning Parameters

MRI experiments were conducted by a 3T MRI scanner (Prisma, Siemens, Erlangen, Germany) with 20-channel head coil at National Taiwan University. During the experiment, a high-resolution T1-weighted anatomical image was initially scanned using the 3D-MPRAGE sequence with 256 × 256 × 192 matrix size; 0.93 × 0.93 × 0.93 mm^3^ resolution; inversion time (TI) = 900 ms; repetition time (TR) = 2,000 ms, echo time (TE) = 2.3 ms; flip angle (FA) = 8°; bandwidth = 200 Hz/pixel; NEX = 1. Total scan time was 6 min 14 sec. The fMRI protocol was using a single-shot gradient-echo echo-planar imaging (GE-EPI) sequence with following imaging parameters: TR = 2 s, TE = 34 ms, FA = 84°, bandwidth = 3,005 Hz/pixel, matrix size = 64 × 64 × 33, and FOV = 210 × 210 mm^2^. Stimuli were presented via E-prime 2.0 (Psychology Software Tools, Pittsburgh, PA, USA) with a back-projection projector in 800 × 600 resolution. Participants viewed the stimuli using a mirror mounted on the head-coil and the viewing field was 8.4° (horizontal) by 6.3° (vertical) at a viewing distance of 420 cm. Participants were instructed to respond with a button press using the index and middle fingers of their right hand (Lumina response pad; Cedrus, San Pedro, CA, USA).

During the rs-fMRI scans, the participants were instructed to stay still with their eyes open, to not fall into sleep and to not think of anything in particular. The rs-fMRI scan contained 180 measurements (6 min) per session. During the AUTs, the participants were instructed to view gray-scale pictures of various objects from daily life (through a projector), and to think of as many alternative usages as possible within a fixed duration (20 s). Each AUT session contained 12 trials, including one easy-level picture, one difficult-level picture, and ten normal-level pictures to balance the difficulty level, with fixation and cues of 6 s in between; within each trial one single picture was presented for 20 sec. During the 20 s of the picture presentation, the participants were instructed to press the button immediately when they thought of a special usage; and the button-press numbers and the response time were recorded. Each AUT task contained 156 scans (5 min 12 s) in total. After the cessation of each AUT, the participants were asked to recall the answers retrospectively, and the answers were manually recorded by the experimenter. During the 0-back tasks, one English letter was displayed on screen for 0.5 s, followed by a cross fixation for 1.5 s. The participants were instructed to respond using their right hand to press the right button when the letter appearing on the screen was “X”; otherwise, they were to press the left button. The 0-back fMRI contained 152 scans (5 min 4 s) in each session. The datasets generated for this study are available on request to the corresponding author.

### AUT Performance

Beyond the creativity assessment obtained through the CAQ, the two creativity indices of AUT (e.g., originality and fluency) were also evaluated for each participant according to the scoring method of previous studies (Hao et al., 2015). Fluency was estimated on the basis of the total number of ideas given during the AUT trials. Originality was estimated on the basis of statistically infrequent responses using the following means. The generated answers from the AUT were collected into a comprehensive lexicon for comparison. Three trained raters independently assessed the originality of the AUT performance for every participant. If 10% or less of the participants in the sample gave the same response then it was given a score of “1,” and all other responses received scores of “0.” The inter-rater agreement was with intra-class correlation coefficient of 0.97. Subsequently, the originality scores of the AUT performance assigned by the three raters were averaged for every participant.

### fMRI Analyses

The data were processed using Analysis of Functional Neuro Images (AFNI) (Cox, 1996). We processed the task data according to the following steps: (a) motion correction; (b) coregistration; (c) smoothing with full width at half maximum (FWHM) = 6 mm, and (d) normalization to the MNI space. During the AUT sessions, participants' responses were recorded and used as the events for divergent thinking, enabling the event-related fMRI analysis. Using the events convolved with the canonical hemodynamic function, we subsequently applied the generalized linear model (GLM) to generate the activation map for each AUT and calculated the average beta map across three AUTs for each individual. The consequent AUT results were equally divided into two groups (*n* = 20 for each of the high- and low-creative groups) according to their CAQ scores (the cut-off score for the CAQ was 8), and a two-sample *t*-test was conducted to generate the contrast maps of AUT brain activity.

The rs-fMRI data were processed according to the following steps: (a) field-map correction; (b) motion correction; (c) coregistration; (d) detrending and filtering (0~0.1 Hz); (e) smoothing with FWHM = 5 mm, and (f) normalization; and (g) the physiological noise removal including white matter, cerebrospinal fluid and six motion parameters. Subsequently, the functional connectivity maps before and after AUT were established through the seed-correlation analysis, targeting on two specific brain networks: (1) the bilateral posterior cingulate cortex (PCC) [±3, −53, 26] for assessing DMN (Van Dijk et al., 2010; Yan et al., 2013), and (2) the seeding at the peak of AUT contrasts between the high- and low-creative groups. Subsequently, the group-based RSFC maps underwent the following statistical analyses to estimate the brain reconfigurations.

### Statistical Analyses

All group analyses of the fMRI results were conducted on the basis of analysis of variance (ANOVA) and *t*-test using AFNI. The group difference in brain activity during AUTs were evaluated through a two-sample *t*-test between the high- and low-creative groups. The two brain networks associated with divergent thinking were compared for RSFC according to the 2 (high- and low-creative) × 2 (pre- and post-AUT) two-way ANOVA (3dMVM), inclusive of the mean framewise displacement to minimize the motion effect on RSFC findings. The multiple comparisons in the group analysis were corrected through the 3dClustSim approach with the auto-correlation function and the significance level was *p* < 0.05 (uncorrected *p* < 0.001, cluster size = 80 mm^3^). To further determine the relationships between creativity scores and the divergent thinking effect of RSFC, the Pearson correlation analysis was used to measure the association between the behavior indices of each individual (originality and fluency) and the RSFC changes (Post-Pre) from selected regions of interested (ROIs). The centers of the spherical ROIs (radius = 5 mm) were placed at the peak value of the significant changes in the contrast maps. The effect of divergent thinking was assessed by calculating the RSFC differences between Pre- and Post-AUT after Fisher's z transformation of the correlation coefficients. The z-transformed RSFC changes from the selected ROIs were correlated with the creativity indices (originality and fluency) with false-discovery rate (FDR) correction across ROI-wise comparisons. The statistical calculations were performed using SPSS 20 (IBM Statistical Package for the Social Sciences).

## Results

### Creativity Performances: CAQ and AUT

The average CAQ score for the 40 participants was 10.8 ± 10.4, ranging from 1 to 62. Regarding the overall AUT performances, the evaluated originality and fluency were 0.33 ± 0.11 and 3.1 ± 1.0, respectively. The CAQ scores were adjusted by the log-transform (Form and Kaernbach, 2018), and the log(CAQ) scores exhibited a positive trend toward the AUT fluency (*r* = 0.30, *p* = 0.06), without prominent relationships with originality (*r* = 0.22, *p* = 0.16) for all participants. To test the difference in brain activity between high and low creativity individuals, the following results were divided into two groups with equal sample size (*n* = 20 for each group); where their CAQ scores were 16.6 ± 12.2 for the high-creative group and 5.0 ± 1.9 for the low-creative group. In addition, the two groups showed no significant differences in age (*p* = 0.06), gender (*p* = 0.53) and motion during the two rs-fMRI scans (framewise displacement: *p* = 0.39).

### Brain Activity Associated With AUT

**Figure 1:**
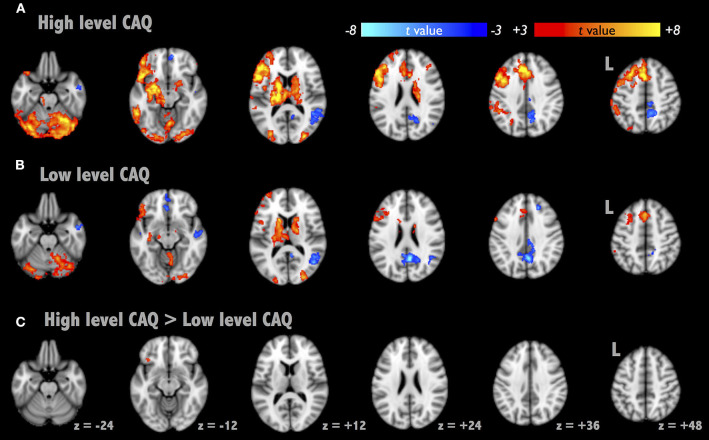
Brain activation maps of Alternative Uses Task (AUT). We presented the activation maps divided into two groups as follows: high- and low-creative [**(A,B)**, respectively, segregated by CAQ scores]. **(C)** The AUT contrast map between the two groups, where the contrast region subsided in the left inferior frontal gyrus (BA 47).

The AUT-associated brain activations were located at the left superior temporal gyrus, anterior cingulate gyrus, declive of the cerebellum, caudate and occipital visual cortex (Figure 1, corrected *p* < 0.01). Figures 1A,B illustrate the average activation maps of AUT for the high-creative and low-creative groups, respectively, where the high-creative group exhibited relatively broad spatial extents in AUT engagements relatively. Negative activations were found lateralized to the right hemisphere, specifically located at the right PCC and right inferior parietal lobule. Figure 1C depicts the contrast between the two groups by independent *t*-test (corrected *p* < 0.05). A difference between the groups was observed only in the left IFG (BA47, [−32, 24, −14]), which was taken as the seed localization for the subsequent RSFC analyses. Supplementary Figure 1 shows the overall AUT activation map across all participants and Supplementary Table 1 lists the detailed description of AUT-related brain activities.

### Functional Connectivity: IFGN and DMN

**Figure 2:**
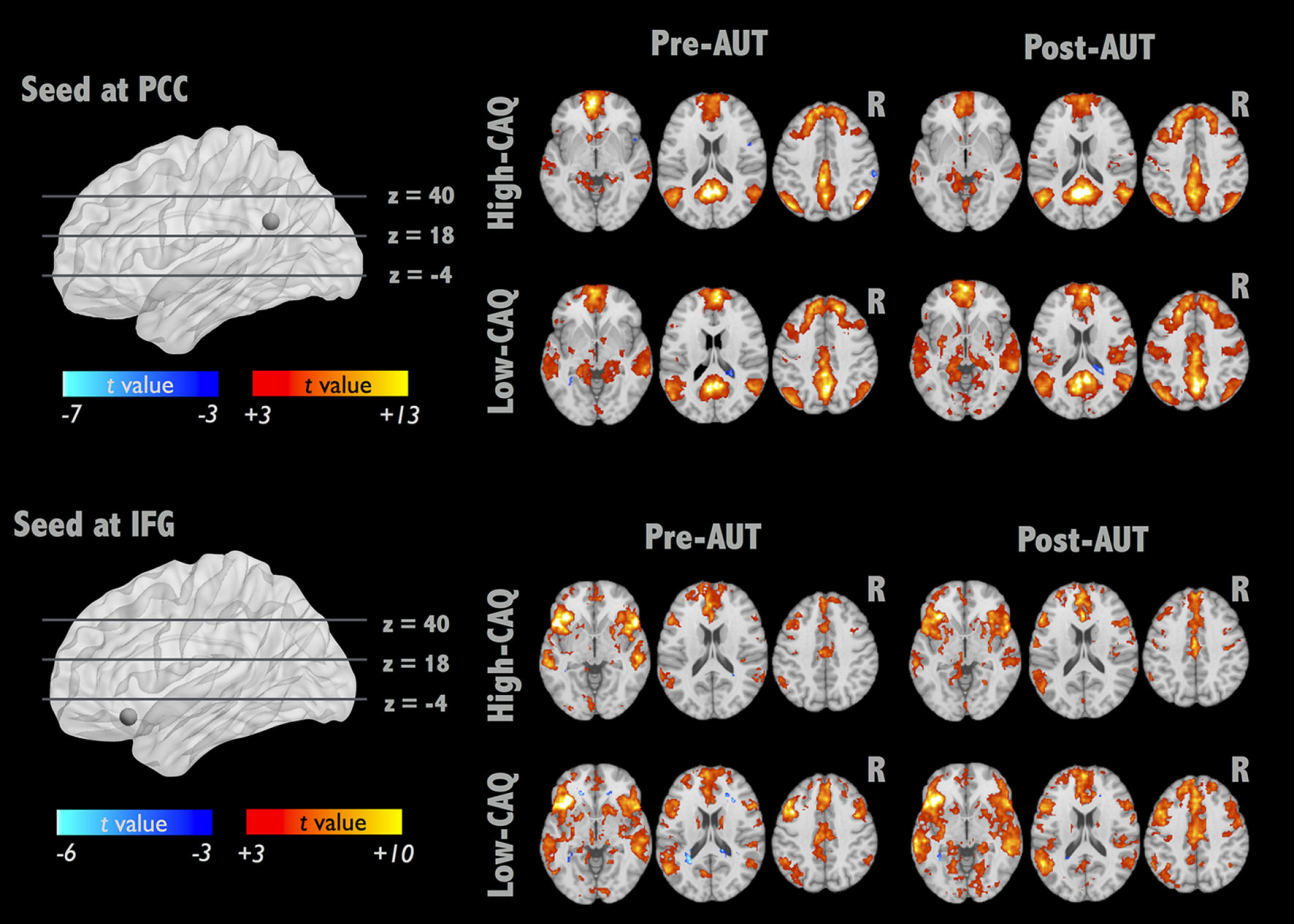
IFGN and DMN RSFC patterns before and after AUT. For each network, the top panel displays the one-sample RSFC map of Pre-AUT and Post-AUT for the high-creative group, and the lower panel refers to the low-creative group.

To further elucidate the alterations of brain integrity over the divergent thinking process, we applied a seed-correlation approach to investigate RSFC. Two brain networks were targeted with the seed locations in bilateral PCC for DMN and left IFG for IFG-associated network (IFGN) to assess the group (high- vs. low-creative) and divergent thinking (Pre- vs. Post-AUT) effects. The upper panel of Figure 2 illustrates the DMN connectivity maps exhibiting strong connections to the bilateral medial and middle frontal, PCC, and bilateral angular gyrus (corrected *p* < 0.01). The lower panel of Figure 2 illustrates the IFGN connectivity maps (corrected *p* < 0.01). The IFGN was associated with the bilateral superior frontal, medial frontal, anterior cingulate, insula, superior, and middle temporal gyri. The detailed information of the group × divergent thinking comparisons (high-creative, low-creative, Pre-AUT and Post-AUT) for IFGN and DMN is listed in Table 1. Prior to the AUT engagements (Pre-AUT), the only significant difference in IFGN between the groups was in the right angular gyrus (AG) and right inferior parietal lobule (IPL), but no group difference was observed in DMN at the baseline level. However, the RSFC of both networks changed after performing the AUT. Table 1 demonstrates that the group difference of RSFC_DMN_ became prominent after AUT engagements, and the RSFC_IFGN_ showed different network reconfigurations between the two groups.

**Table 1:** Group comparison of IFGN and DMN RSFC before and after AUT.

**Brain area**	***t*-value**	**Voxel size**	**Peak x**	**Peak y**	**Peak z**
**DMN (SEED AT LEFT INFERIOR PARIETAL GYRUS [±3**, **−53, 26])**					
**Pre-AUT: High-creative** **>** **Low-creative**					
– –					
**Post-AUT: High-creative** **>** **Low-creative**					
Postcentral gyrus (right)	−0.31	41	54	−26	20
Inferior frontal gyrus (right)	−0.34	30	46	34	2
Thalamus (right)	0.24	15	4	−36	10
Middle temporal gyrus (left)	−0.26	12	−46	0	−24
Superior temporal gyrus (left)	−0.31	12	−44	4	−8
**High-creative group: Post-AUT** **>** **Pre-AUT**					
– –					
**Low-creative group: Post-AUT** **>** **Pre-AUT**					
– –					
**IFGN (SEED AT LEFT INFERIOR PARIETAL GYRUS [-32, 24**, **−14])**					
**Pre-AUT: High-creative** **>** **Low-Creative**					
Angular gyrus (right)	−0.28	71	42	−78	28
Inferior parietal lobule (right)	−0.28	56	40	−62	40
**Post-AUT: High-creative** **>** **Low-creative**					
Middle frontal gyrus (right)	−0.26	76	52	2	44
Anterior cingulate cortex (left)	−0.21	25	−10	22	−10
**High-creative group: Post-AUT** **>** **Pre-AUT**					
Inferior parietal lobule (left)	0.18	11	−40	−66	38
**Low-creative group: Post-AUT** **>** **Pre-AUT**					
Inferior occipital gyrus (right)	0.18	28	36	−80	−8
Parahippocampal gyrus (left)	0.2	9	−6	−36	0

### Association Between Functional Connectivity and Creativity Scores

**Figure 3:**
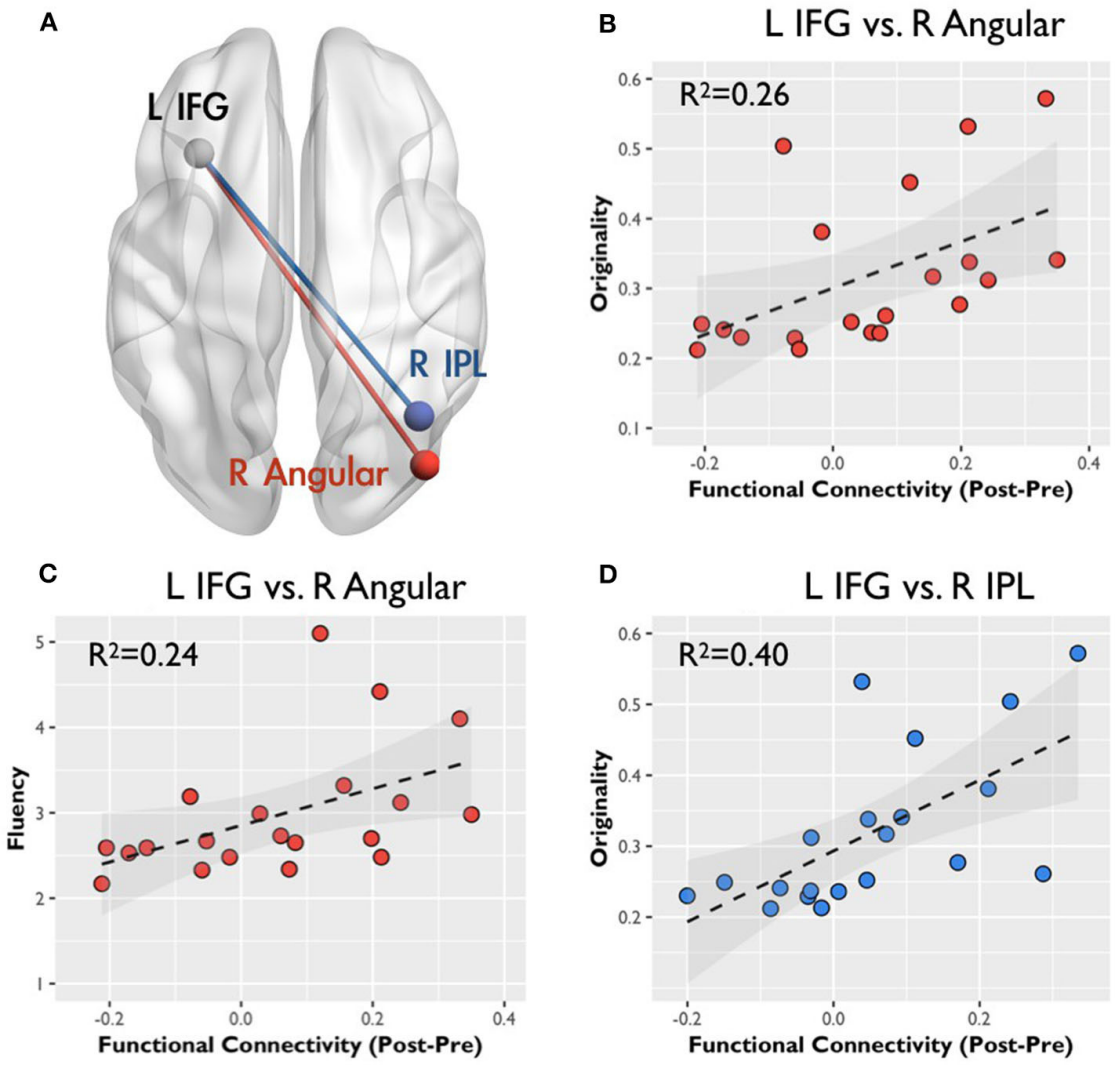
Correlation between the functional connectivity and creative behavior: color regions of two connectivity indicate spatial locations. **(A)** The gray spheres indicate the seeds for functional connectivity, the red line represents the connectivity between the left inferior frontal gyrus (IFG) and the right angular gyrus (AG), and the blue line indicates the connectivity between the left IFG and the right inferior parietal lobule (IPL). All scatter plots display the correlation between RSFC changes (in *z*-value) and CAQ, originality, and fluency scores in r squares. **(B,C)** Represent the relation with the connectivity change between the left IFG and the right AG, and **(D)** correspond to the relation with the connectivity change between the left IFG and the right IPL.

An ROI analysis was conducted to determine the relationship between RSFC changes and creativity scores. The spherical ROIs were placed according to the results in Table 1. Figure 3 presents the RSFC changes between Pre-AUT and Post-AUT and their relationships with AUT originality and fluency in both groups. The RSFC changes in IFGN at the right AG and right IPL were positively correlated with the AUT performances: originality and fluency (originality-ΔFC_IFG−AG_: *r* = 0.51, *p* < 0.02; fluency-ΔFC_IFG−AG_: *r* = 0.49, *p* < 0.03; originality-ΔFC_IFG−IPL_: *r* = 0.63, *p* < 0.001) in the high-creative group, but the correlations in the low-creative group were non-significant. The Steiger's *Z*-test presented significant group differences in the correlation coefficients of originality-ΔFC_IFG−AG_ (*p* < 0.001), fluency-ΔFC_IFG−AG_ (*p* < 0.004) and originality-ΔFC_IFG−IPL_ (*p* < 0.001). Supplementary Table 2 summarizes all correlations between RSFC and creativity assessments [log(CAQ), AUT fluency and originality].

## Discussion

We demonstrated that the divergent thinking process (e.g., AUT) could modulate the RSFC of DMN and IFGN, which were associated with creativity performance. The results indicated that the baseline RSFC_DMN_ networks (Pre-AUT) were similar between the two groups, but the baseline RSFC_IFGN_ could reflect the group difference. Then, the IFGN presented significant network reorganization while the DMN did not reorganize after AUT engagements. Intriguingly, such IFGN reconfiguration was associated with AUT performances (originality and fluency, Figure 3). These findings supported that the dynamic changes of intrinsic network connections could reflect the cognitive performances of divergent thinking. However, the high- and low-creative groups demonstrated distinct network—creativity associations as follows: positive relationships were evident between originality and RSFC changes in the high-creative group but the low-creative group showed non-significant correlations (Supplementary Table 2), further indicating the dissimilar functional architectures involved in divergent thinking among individuals with diverse creativity achievements. These findings suggest that the RSFC_IFGN_ can change dynamically in accordance with the divergent thinking process, and the populations with various creativity levels may employ distinct reconfigurations after divergent thinking.

### Network Reconfiguration After Divergent Thinking

The integrity of brain network can be modulated by the previous cognitive engagements within minutes, and the functional re-organization may be important to the cognitive performances. Previous research investigated the correlation between the IFG connectivity and the subsequent memory (Stevens et al., 2010), disclosing the connectivity facilitates the recognition accuracy. In the current study, we focused on the dynamic RSFC changes of the two networks, DMN and IFGN, corresponded to the networks related with high-creative thinking ability reported in literature (Beaty et al., 2018). Interestingly, the same study also demonstrated the evidence of dynamic RSFC alterations following divergent thinking. In their supporting information, Beaty et al. presented fairly similar correlations between network strengths and creativity scores based on a resting-state dataset (*r* = 0.13 for high-creative networks and *r* = 0.11 for low-creative networks). However, the RSFC–creativity correlation became elevated for high-creative networks when performing the AUT (*r* = 0.35 and 0.28 for two separate datasets), but the correlation turned out non-significant for low-creative networks during AUT engagements (*r* = 0.02 to −0.04 for two separate datasets). The evidence implies that dynamic network reorganizations in AUT can be associated with the creativity performances. In this study, assuming that the short-term network reorganizations induced by AUT could sustain after the task cessation (the second resting state in Post-AUT), we demonstrated that dynamic RSFC alterations were associated with the AUT performance (originality and fluency). Specifically, we prescribed the ROIs from both the creativity trait effect (High-creative > Low-creative defined by CAQ in Table 1) and the creativity state effect (Post-AUT > Pre-AUT), because the CAQ (trait) and AUT performances (state) were highly correlated within each group (Supplementary Table 2). Our results showed that DMN demonstrated the trait difference between groups; however, no prominent state effect was found in DMN. Relatively, IFGN contained both trait and state effect (Table 1), indicating that the IFGN indeed presented the network reconfiguration after AUT engagements. The concept of network reconfiguration was supported by Wei et al. (2014), in which they demonstrated that the RSFC strengths was modulated in general after the performance of a cognitive stimulation task. The dynamic alterations of RSFC following cognitive tasks can be regarded as a short-term functional reconfiguration of brain circuits to facilitate associated cognitive tasks, which fits the neurophysiological perspective of the dynome framework (Kopell et al., 2014). For example, Wang et al. presented the dynamic reorganizations of DMN during and after a word-picture matching task, so as the corresponding whole-brain small-world topology (Wang et al., 2012). However, previous studies did not specifically present the relationship between the network reconfiguration and the cognitive performances. In the current work, Figure 3 illustrates that dynamic IFGN reconfigurations were in accordance with the creativity performances, supporting the short-term reconfiguration and sustenance of network integrity underlying the mental process of divergent thinking.

### Regional Functionality in Divergent Thinking

Three brain regions in the left hemisphere were reported to be involved in divergent thinking—the IFG, pre-/post-central gyri, and the MTG (Boccia et al., 2015), and these are similar to our results of brain activity in AUT. Generally, the lateralization of brain function in the left hemisphere in AUT is considered to be the verbal processing and semantic memory (Thompson-Schill, 2003). In addition, the left hemisphere executes the functions of interpreting and reasoning about the sentences and the causal relationships (Marinsek et al., 2014). By presenting the CAQ-based group difference of brain activation in AUT (Figure 1), the left IFG was highlighted in the performance of creativity, regarded as the functional localizer for the following RSFC analysis.

Figure 3 illustrates the relationship between originality and the corresponding RSFC changes after AUTs; the RSFC changes are prominent between the left IFG, right AG, and right IPL. Previously, the SPL/IPL was the core for top-down attention or abstract thinking (Shomstein, 2012), and the IPL was determined to usually play roles involving the voluntary orienting of attention to a location (Corbetta et al., 2000). In addition, the left IFG and right IPL involved the verbal working memory (LaBar et al., 1999), and the strength of RSFC between the left IFG and right IPL decreased in high working memory loading (Liu et al., 2017). Furthermore, a previous study reported that the low working memory loading task could help the divergent thinking and (Baird et al., 2012). For the multifunction of AG, the right side was determined to play the role of spatial cognition for perceptual learning and shifting attention to relevant information (Seghier, 2013). Therefore, our result demonstrated that the high-creative group leveraged from the frontoparietal reorganizations for elevating the abstract thinking, verbal working memory or spatial cognition involved in AUT. In contrast, the changes of RSFC in the low-creative group showed no relationship associated with the creative indexes. Following the finding, our results revealed the possibility that the low-creative group might adopt distinct strategies with respect to the semantic or sensory processing in AUT.

As evident in Supplementary Table 1, the majority of AUT-induced negative activations were located in the right hemisphere (the superior temporal gyrus, MTG, medial frontal gyrus, precuneus, precentral gyrus, and superior frontal gyrus) in both groups. Previously, the mechanism of the negative activation may have originated from the neural inhibition hypothesis, the affected neurotransmitter caused the reduction of local cerebral blood flow or the elevation of the cerebral metabolic rate of oxygen consumption in the cerebrovasculature (Sten et al., 2017). Interestingly, Benedek et al. also presented similar lateralized negative activation during idea generation (Benedek et al., 2014). To further verify the role of negative AUT activations in creativity, the negative brain activations were associated with the log-transform CAQ, originality, and fluency in both the high- and low-creative groups. We noted significantly positive correlations between the log(CAQ) and the right MTG [52, −10, −16] activity (*r* = 0.52, *p* < 0.02) in the high-creative group, as well as a significantly positive relationship between originality and the right MTG activity (*r* = 0.51, *p* < 0.02). However, the association between the negative activation and creativity performances is beyond the scope of the current work. Future studies are warranted to discern the underlying mechanism of divergent thinking.

### Disparity Between High- and Low-Creative Groups

About grouping of CAQ scores, we chose the median split because of a positively-skewed distribution of CAQ in Carson's report. Additionally, the average CAQ score of Carson's report was as high as 14.4, the cut-off line would be around 8 to 9 when we adopted the median split (Carson et al., 2005). Therefore, the cut-off threshold of 8 points was in agreement with Carson's report. Although we used the CAQ (the creative traits) for splitting participants into two groups, we confirmed the functional brain distinction between the high- and low-creative groups when performing AUT (Figure 1), revealing the active role of the left IFG in AUT engagements. By definition, the participants with high CAQ scores were believed to possess the capability of openness to experience (Carson et al., 2005), in part explaining that the high-creative group uses the brain systems of abstract thinking to achieve superior originality. Moreover, the between-group comparison disclosed that the DMN connectivity did not exhibit significant differentiation at the baseline level before AUT engagements (Table 1). In IFGN, the baseline connectivity strength between the left IFG and right IPL in the high-creative group was higher than that in the low-creative group at the Pre-AUT condition. This finding was contradictory to the previous report (Beaty et al., 2014). The disparity might be originated from the cultural difference, because previous study showed that frontal-parietal attentional control network involves in the visual and attention task and these functions exhibits cultural disparity (Han and Northoff, 2008; Hedden et al., 2008). Then, the connection from IFG to IPL involved the lexical judgment in Chinese studies (Deng et al., 2012). Future studies are warranted to prove such conjecture. In addition, when comparing the RSFC_IFGN_ changes between the two groups in Table 1, the low-creative group demonstrated more Pre-Post connectivity differences in the posterior brain, whereas the high-creative group mostly remained unchanged. Wei et al. observed similar between-group differences in RSFC between MTG and MPFC (Wei et al., 2014). They suggested that the group with higher TTCT scores exhibited fewer RSFC differences after creativity training, though the RSFC changes were not associated with the creativity performances. Collectively, it is speculated that each individual may possess specific brain-network plasticity to facilitate the performances of divergent thinking, where such brain reconfigurations are distinct between the high- and low-creative groups.

### Limitation

This study has several limitations. First, the sample size of the current study was above the request of 36 samples for sufficient fMRI replicability (Turner et al., 2018); however, the statistical power might be reduced after splitting the samples into two equal-size groups. Compared with literature, the AUT-induced brain activities were in well-agreement with previous findings, but the RSFC might be inconsistent with other studies. The sample size higher than 20 in each group is recommended for future neuroimaging studies with AUT engagements. Second, we observed only the two most addressed functional networks in AUT, namely DMN and IFGN, by which we might miss additional AUT-related brain reconfigurations in other brain networks, such as the reported global architectures involved in divergent thinking (Beaty et al., 2018). Future studies are warranted to test the whole-brain functional changes other than DMN and IFGN. Third, the group separation (based on CAQ) and their creative performances (i.e., originality and fluency) were all defined by the subjective preferences from the participants or the raters. However, creativity judgment is a relatively challenging task due to the lack of objective definitions, as aforementioned. Therefore, we used the AUT activity to verify the effectiveness of group separation and employed three raters to minimize the subjectivity involved in the creativity scoring. Fourth, we did not include any other cognitive tasks to test whether such network reconfiguration is specific to the divergent thinking. It was unanswered because the assumption that the network plasticity is subject to distinct cognitive tasks has yet been studied systematically. The post-cognition network plasticity is pending for further investigations in the near future. At last, the causal relation between the network reorganization and creativity performance remains limited, because the findings in this study was built upon the assumption that the AUT-associated network reconfigurations can sustain in a short period of time after the cessation of tasks. The short-term sustenance of functional organizations in brain circuits shall be further tested before confirming the causal relationship between network plasticity and creativity.

## Conclusion

Using multiple AUTs for creativity engagements, we presented the prolonged changes of RSFCs (DMN and IFGN) correlated with the performances of divergent thinking. Furthermore, individuals with different creativity level (high- and low-creative groups) might present diverse alterations of RSFC changes. Before divergent thinking, both high- and low-creative group did not exhibit significant difference of DMN connectivity, but the group difference was highlighted after AUT engagements. Meanwhile, the IFGN indeed presented the network reconfiguration after divergent thinking. Furthermore, the ΔFC_IFG−AG_ and ΔFC_IFG−IPL_ positively contributed to the AUT performances in the high-creative group, but no prominent brain-behavior relation was found in the low-creative group. These findings indicated that divergent-thinking performances could be modulated by distinct creativity traits and diverse brain-network reconfigurations.

## Data Availability Statement

The datasets generated and analyzed in the current study are not publicly available due to IRB but are available from the corresponding author on reasonable request. Requests to access the datasets should be directed to Changwei W. Wu, sleepbrain@tmu.edu.tw.

## Ethics Statement

The studies involving human participants were reviewed and approved by Research Ethics Committee of National Taiwan University. The patients/participants provided their written informed consent to participate in this study.

## Author Contributions

H-YW: data collection, analysis, and writing. B-CK and C-MH: experiment design and writing. P-JT and A-LH: analysis and writing. L-MH: analysis. C-YL: data collection. J-HC: suggestion of writing. CW: the main idea, experimental design, and writing. All authors contributed to the article and approved the submitted version.

## Conflict of Interest

The authors declare that the research was conducted in the absence of any commercial or financial relationships that could be construed as a potential conflict of interest.
