# Supplementary material for: Think Hard or Think Smart: Network Reconfigurations After Divergent Thinking Associate With Creativity Performance
Source: Front Hum Neurosci. 2020 Nov 20;14:571118. doi: 10.3389/fnhum.2020.571118 (PMC7714934; doi:10.3389/fnhum.2020.571118)
Supplement: Supplementary file 1 [file Table_1.pdf]

Table S1 List of activated brain regions in alternative uses task (AUT): comparison between both high- and low-creative groups, and the contrast between two groups. (Coordinate in MNI space with marks of Yeo's 17 networks)

Brain area	Yeo's network	*t*-value	Voxels	X	Y	Z
**AUT: high-creative group**						
Inferior Frontal Gyrus (BA 47)	17	6.44	21200	-50	18	-10
Declive	--	6.83	13084	32	-70	-26
Cingulate Gyrus	11	-4.02	1014	4	-52	42
Inferior Parietal Lobule	12	4.08	793	-54	-40	54
Superior Temporal Gyrus	14	-5.12	549	54	-44	12
Superior Parietal Lobule	13	4.26	122	-32	-72	54
Middle Frontal Gyrus	12	4.34	100	42	38	26
Inferior Parietal Lobule	7	3.95	96	-56	-38	24
Middle Temporal Gyrus	17	-4.82	79	52	-10	-16
Postcentral Gyrus	4	4.53	78	-64	-20	14
Inferior Frontal Gyrus	17	6.19	61	52	38	-6
Medial Frontal Gyrus	16	-4.68	58	4	52	-12
**AUT: low-creative group**						
Inferior Frontal Gyrus (BA 47)	14	4.54	10627	-54	14	-8
Precuneus	16	-7.40	1365	4	-54	36
Cingulate Gyrus	8	5.72	1289	0	18	42
Superior Temporal Gyrus	14	-4.00	549	56	-48	16
Middle Temporal Gyrus	17	-4.52	208	52	-16	-14
Precentral Gyrus	3	-4.09	138	44	-18	62
Medial Frontal Gyrus	16	-4.33	119	0	56	-6
Middle Frontal Gyrus	8	4.36	108	-36	56	12
Anterior Cingulate Cortex	16	-4.62	100	-2	40	-6
Inferior Parietal Lobule	12	4.91	100	-50	-44	56
Middle Frontal Gyrus	12	4.02	90	-46	32	28
Superior Frontal Gyrus	16	-3.90	84	30	40	46
Middle Temporal Gyrus	17	-4.14	49	-50	-10	-12
**AUT: Contrast between high-CAQ and low-CAQ group**						
Inferior Frontal Gyrus	17	4.62	45	-36	24	-14
