# Supplementary material for: Think Hard or Think Smart: Network Reconfigurations After Divergent Thinking Associate With Creativity Performance
Source: Front Hum Neurosci. 2020 Nov 20;14:571118. doi: 10.3389/fnhum.2020.571118 (PMC7714934; doi:10.3389/fnhum.2020.571118)
Supplement: Supplementary file 2 [file Table_2.pdf]

Table S2: Pearson correlation coefficients between behavior score and RSFC of DMN (Post-Pre)

	log(CAQ)	Originality	Postcentral Gyrus (R)	Inf. Frontal Gyrus (R)	Thalamus (R)	Mid. Temporal Gyrus (L)	Sup. Temporal Gyrus (L)
High-CAQ group							
log(CAQ)	1		0.24	0.18	0.07	-0.16	0.28
Originality	**0.46***	1	0.14	0.15	0.15	-0.16	0.09
Fluency	**0.58****	**0.78****	0.16	0.19	0.18	-0.07	0.20
Low-CAQ group							
log(CAQ)	1		0.02	0.14	-0.28	0.01	0.23
Originality	0.42$^{\#}$	1	-0.27	-0.35	-0.16	-0.08	-0.18
Fluency	**0.66****	**0.78****	-0.03	-0.05	-0.30	0.18	-0.02

* $p < 0.05$, ** $p < 0.01$, $^{\#}$ Marginal significance $0.08 < p < 0.05$

Pearson correlation coefficients between behavior score and RSFC of IFGN (Post-Pre)

	log(CAQ)	Originality	Inf. Occipital Gyrus (R)	Parahippocampal Gyrus (L)	Inf. Parietal Lobule (L)	Angular Gyrus (R)	Inf. Parietal Lobule (R)	Mid. Frontal Gyrus (R)	Anterior Cingulate Cortex (L)
High-CAQ group									
log(CAQ)	1		-0.37	0.22	0.21	**0.47***	0.35	-0.01	-0.18
Originality	**0.46***	1	-0.16	0.23	0.43$^{\#}$	**0.51***	**0.63****	0.14	-0.11
Fluency	**0.58****	**0.78****	-0.33	0.34	0.38	**0.49***	0.36	0.04	0.04
Low-CAQ group									
log(CAQ)	1		-0.08	0.07	0.03	0.03	-0.20	-0.28	0.11
Originality	0.42$^{\#}$	1	-0.14	-0.32	-0.21	-0.30	-0.17	-0.31	0.36
Fluency	**0.66****	**0.78****	0.16	-0.16	0.03	-0.14	-0.18	-0.17	0.07

* $p < 0.05$, ** $p < 0.01$, $^{\#}$ Marginal significance $0.08 < p < 0.05$
