# Supplementary material for: Think Hard or Think Smart: Network Reconfigurations After Divergent Thinking Associate With Creativity Performance
Source: Front Hum Neurosci. 2020 Nov 20;14:571118. doi: 10.3389/fnhum.2020.571118 (PMC7714934; doi:10.3389/fnhum.2020.571118)
Supplement: Supplementary file 3 [file Image_1.pdf]

Supplementary Information

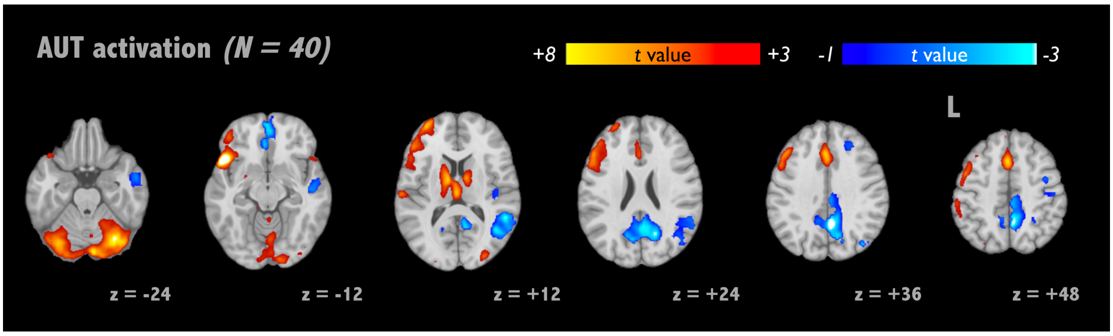

Figure S1 Brain activation maps of Alternative Uses Task (AUT) for all participants (N = 40, corrected $p < 0.01$).
